# Refractory esophageal anastomotic stricture after esophageal atresia surgery improved with retrograde balloon dilatation through gastrostomy followed by laparoscopic fundoplication: a case report

**DOI:** 10.1186/s40792-023-01754-0

**Published:** 2023-09-22

**Authors:** Yoichi Nakagawa, Hiroo Uchida, Akinari Hinoki, Chiyoe Shirota, Wataru Sumida, Satoshi Makita, Kazuki Yokota, Hizuru Amano, Akihiro Yasui, Daiki Kato, Yousuke Gohda, Takuya Maeda

**Affiliations:** 1 Department of Pediatric Surgery, Nagoya University Graduate School of Medicine, 65 Tsurumai-cho, Showa-ku, Nagoya, Aichi 466-8550 Japan; 2 Department of Rare/Intractable Cancer Analysis Research, Nagoya University Graduate School of Medicine, 65 Tsurumai-cho, Showa-ku, Nagoya, Aichi 466-8550 Japan

**Keywords:** Anastomotic stricture, Balloon dilatation, Esophageal atresia

## Abstract

**Background:**

An esophageal anastomotic stricture (EAS) after an esophageal atresia surgery occurs in approximately 4–60% of the cases, and its first-line therapy includes balloon dilatation. Oral balloon dilatation cannot be performed in some EAS cases; conversely, even if dilatation is possible, these strictures recur in some cases, necessitating a surgical procedure for repairing the stenosis. However, these procedures are invasive and have short- and long-term complications. If an EAS recurs repeatedly after multiple balloon dilations, gastroesophageal reflux disease (GERD) may be the underlying cause. A fundoplication procedure may be effective for treating a refractory EAS, as in the present case.

**Case presentation:**

A neonatal patient with type D esophageal atresia underwent thoracoscopic esophago-esophageal anastomosis at the age of 1 day, and her postoperative course was uneventful. Thereafter, the patient underwent gastrostomy for poor oral intake at the age of 3 months. After gastrostomy, the patient presented with a complete obstructive EAS. Balloon dilatation via the oral route was attempted; however, a guidewire could not be inserted into the EAS site. Hence, retrograde balloon dilatation via gastrostomy was performed successfully. However, the EAS recurred easily thereafter, and laparoscopic anti-reflux surgery was performed to prevent GERD. The anti-reflux surgery cured the otherwise refractory EAS and prevented its recurrence.

**Conclusions:**

Retrograde balloon dilatation is another treatment option for an EAS. When an EAS recurs soon after dilatation, the patient must be evaluated for GERD; if severe GERD is observed, an appropriate anti-reflux surgery is required before dilating the EAS.

## Background

An esophageal anastomotic stricture (EAS) after an esophageal atresia (EA) surgery occurs in approximately 4–60% of the cases [1–3], and its first-line therapy includes balloon dilatation [4]. When balloon dilatation is impossible to perform or when patients are refractory despite multiple balloon dilatations, surgery is essential; the surgical procedures for treatment include EAS site resection with esophago-esophageal anastomosis, gastric pull-up, and interpositioning of the colon and jejunum. However, these procedures are invasive and have short- and long-term complications. Herein, we report a case of a refractory, complete obstructive EAS that occurred after a type D EA surgery involving gastrostomy and balloon dilatation. To preserve the native esophagus, the patient was treated with retrograde balloon dilatation via gastrostomy followed by laparoscopic fundoplication.

## Case presentation

**Fig. 1:**
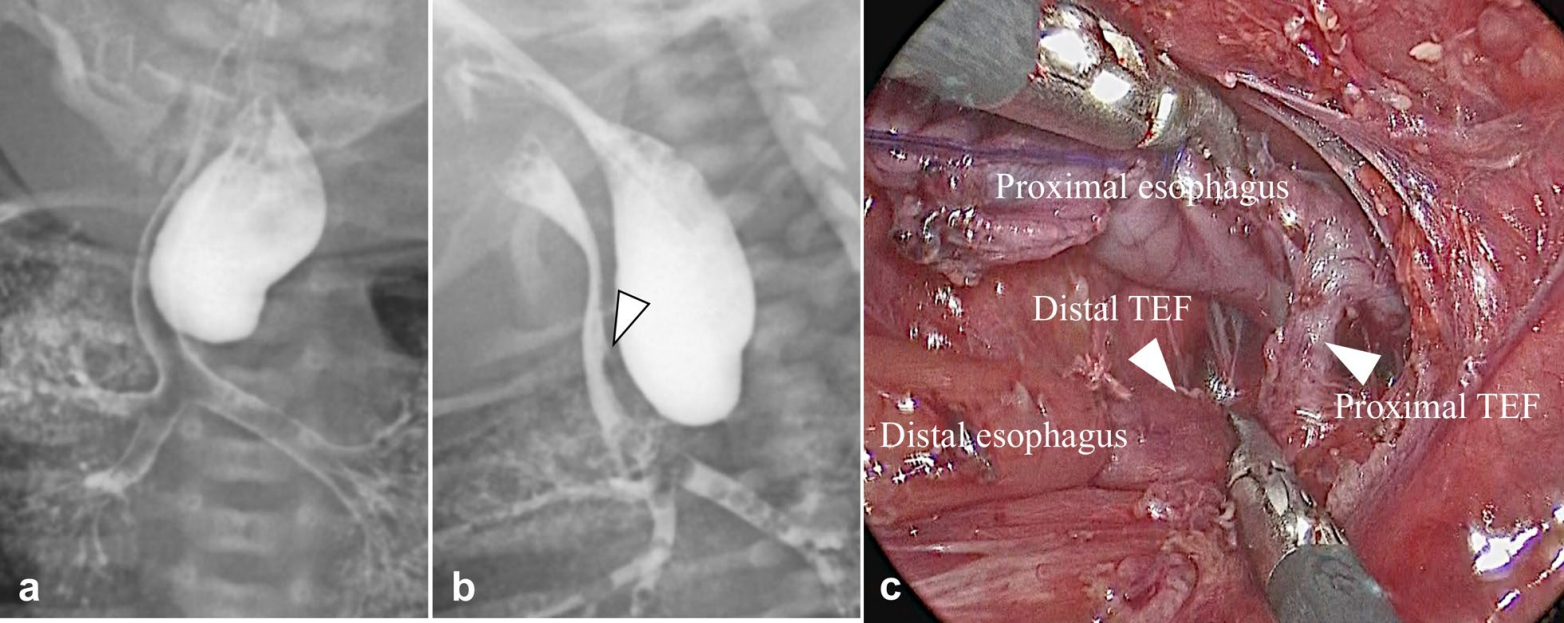
Preoperative and operative findings. PA view (**a**) and lateral view (**b**) during contrast examination suggest the presence of a TEF in the proximal esophagus (white arrowhead). Intraoperative findings show two TEFs at the proximal and distal esophagus (arrowhead), resulting in type D esophageal atresia (**c**). PA: posterior anterior; TEF: tracheoesophageal fistula

The patient was a second-born, monochorionic, diamniotic twin with a gestational age of 36 weeks and 2 days. The patient weighed 2054 g and had Apgar scores of 1 and 4 at 1 and 5 min, respectively. The prenatal course was uneventful, with no apparent prenatal diagnosis. However, the patient was referred to our hospital with suspected EA. We routinely perform contrast examinations [5], and a contrast examination on the day of admission revealed a tracheoesophageal fistula at the proximal esophagus. Intraoperative findings confirmed the diagnosis of type D EA. Thoracoscopic esophago-esophageal anastomosis with ligation of two parts of the tracheoesophageal fistula was performed at the age of 1 day (Fig. 1). The postoperative course was uneventful except for tracheomalacia and dysphagia, and enteral nutrition was started via a nasogastric tube at the age of 6 days. A contrast study on postoperative day 21 revealed an EAS and gastroesophageal reflux with sliding hernia. Mosapride citrate, rikkunshito, and famotidine were administered to prevent gastroesophageal reflux. Tracheomalacia was well-controlled with continuous positive airway pressure. The cause of dysphagia was unknown. Soon after the operation, left recurrent laryngeal nerve palsy was noted; however, it improved at 3 weeks postoperatively. Dysphagia persisted despite this; hence, nutrition was continued via a nasogastric tube. The patient was transferred to a local hospital at the age of 56 days.

At the local hospital, the patient developed aspiration pneumonia and underwent tracheal intubation at the age of 65 days. In addition, the patient had poor oral intake and was fed using a nasogastric feeding tube. The feeding tube was accidentally removed, and re-insertion was difficult due to the EAS; hence, gastrostomy was performed for enteral feeding at the age of 3 months. However, severe gastroesophageal reflux disease (GERD) remained unresolved after the procedure, and the EAS progressed and became almost obstructive. The patient’s condition could not be controlled at the local hospital; therefore, she was re-transferred to our institution.

**Fig. 2:**
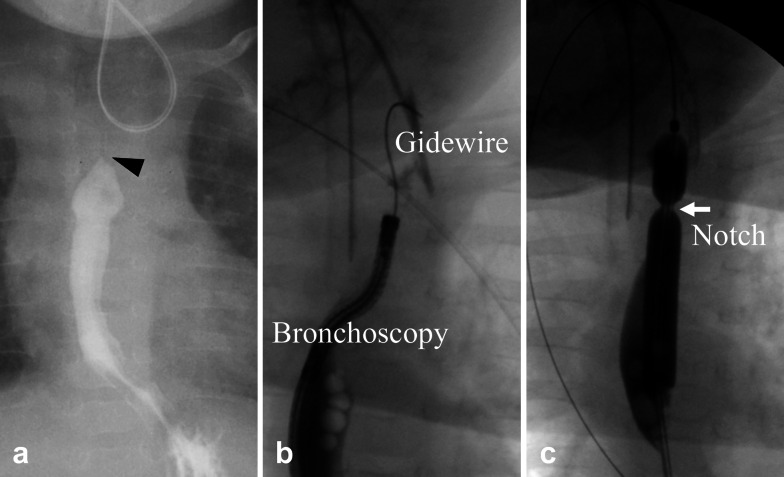
Contrast examination at retrograde balloon dilatation. Contrast examination at the previous hospital shows complete obstruction at the anastomosis site (**a**; black arrowhead). A guidewire is inserted via the gastrostomy site under bronchoscopic guidance (**b**). Retrograde balloon dilatation is performed until 7 mm dilatation and a severe anastomotic stricture is noted as white arrow (**c**)

**Fig. 3:**
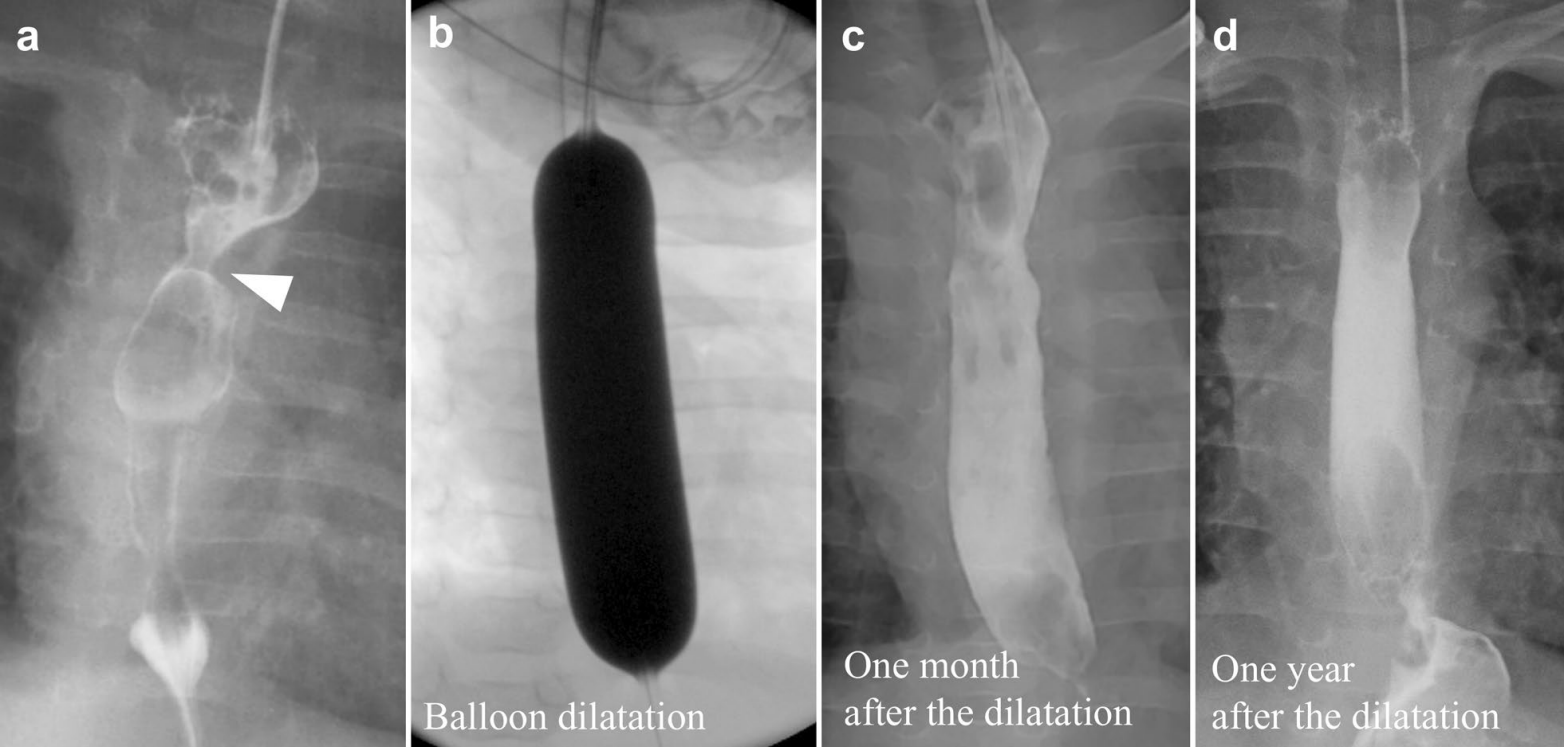
Contrast examination after the fundoplication. Contrast examination after fundoplication shows an anastomotic stricture (**a**), which was improved after balloon dilatation (**b**). Contrast examinations performed 1 month (**c**) and 1 year after dilatation (**d**) show no apparent anastomotic strictures

**Fig. 4:**
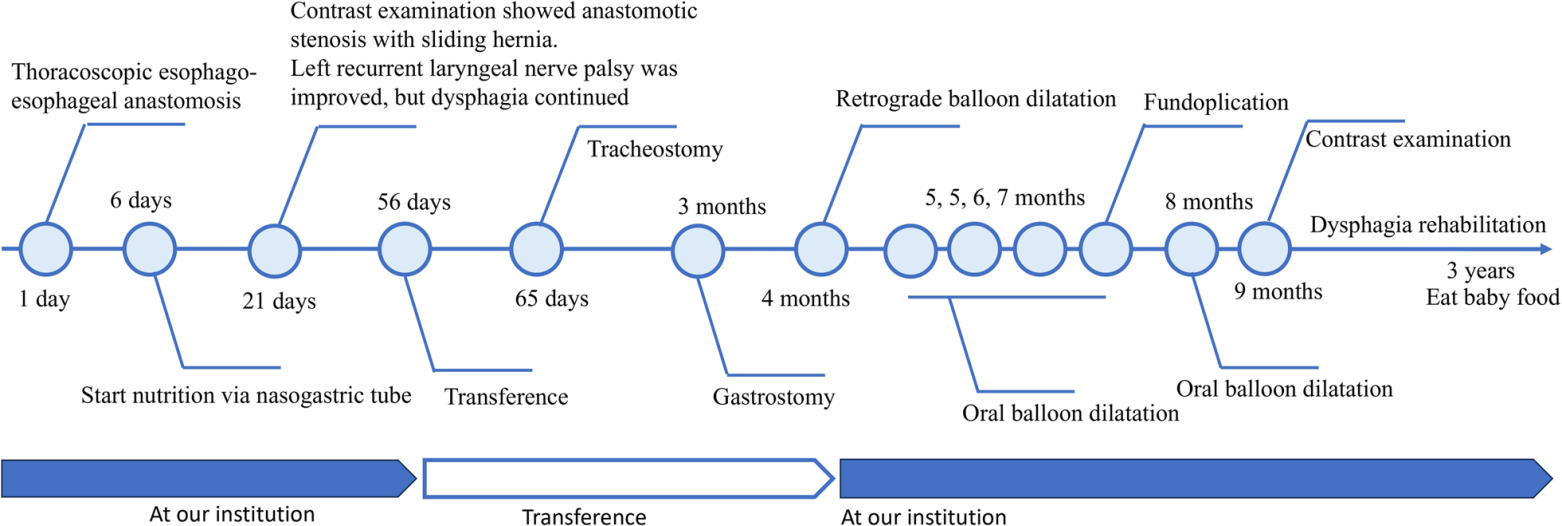
Flow chart of the treatment

Contrast examination at the local hospital revealed a complete obstruction at the anastomosis site (Fig. 2). Upper endoscopy was performed to evaluate the EAS site, which revealed a complete obstruction that looked like a membrane. We unsuccessfully attempted to insert a 0.025-inch Jagwire™ Plus (Boston Scientific, Tokyo, Japan) via the oral route blindly under fluoroscopic view. Retrograde balloon dilatation for the EAS was performed through the gastrostomy site at the age of 4 months (Fig. 2). Briefly, by advancing a 2.8 mm bronchoscope and a guidewire through the gastrostomy site, a 0.025-inch Jagwire™ was easily inserted through the EAS site under fluoroscopic and bronchoscopic guidance. CRE™ wire-guided balloon dilatation catheters (Boston Scientific, Tokyo, Japan) were then inserted through the Jagwire™ via the gastrostomy site, and the EAS site was dilated until 7 mm. After retrograde balloon dilatation, balloon dilatation via the oral route became possible. Oral balloon dilatation with proton pump inhibitor treatment was performed four times, twice at the age of 5 months and once at the ages of 6 and 7 months each; however, the EAS recurred easily after these procedures. In addition, after improvement in the EAS by retrograde balloon dilatation, the patient began vomiting frequently. GERD was considered the cause of the repeated re-strictures; hence, laparoscopic Toupet fundoplication was performed at the age of 7 months. The esophagus was gently separated from the diaphragmatic crus to avoid causing esophageal dysmotility after surgery. The intra-abdominal esophagus was mobilized for at least 3 cm without strong traction. The diaphragmatic hiatus was sutured to prevent hiatal hernia. Because the patient had undergone gastrostomy and the stomach volume was relatively small, a wrap was difficult to make; however, a tension-free wrap was possible due to Toupet fundoplication. The first balloon dilatation after fundoplication was performed at the age of 8 months, yielding an almost complete improvement in the anastomotic stricture. The second balloon dilatation after fundoplication was performed during the final check at the age of 9 months, and contrast examination revealed no anastomotic strictures. The anastomotic stricture did not recur for 3 years and 7 months after the last dilatation (Fig. 3). However, dysphagia persisted after fundoplication. Laryngoscopy revealed no apparent recurrent laryngeal nerve palsy and laryngeal movement; however, the patient drank a little and refused to eat. Rehabilitation for dysphagia was continued, and the patient drank a cup of tea without aspiration at the age of 1 year and 5 months and started to eat solids at the age of 1 year and 10 months. The patient started eating baby food at the age of 3 years. Dysphagia has improved gradually; we believe its etiology is an unexplained developmental delay in the patient. To better depict the timeline of the case, a flow chart of the treatments provided has been given in Fig. 4.

## Discussion

The risk factors for an EAS include long-gap EA, postoperative anastomotic leakage [2, 6], trans-anastomotic tube placement [7], and GERD [2]. Balloon dilatation is the first-line treatment for an EAS [4]. To avoid a dilator tip directly perforating the esophagus, a guidewire is inserted, and its passage and position are evaluated using endoscopy or fluoroscopy. However, in case of a severe EAS (i.e., a complete obstructive EAS), it is impossible to insert a guidewire orally. In our case, the patient had a complete obstructive EAS at the time of transfer. Gastrostomy feeding blinded the EAS-related impaired esophageal clearance, while the complete obstructive EAS blinded the GERD symptoms; these were assumed to result in delayed recognition of the complete obstruction in the former hospital. Retrograde balloon dilatation (i.e., insertion of a guidewire via a gastrostomy site) is another option for EAS treatment [8] in patients with gastrostomy, in whom insertion of a guidewire via the oral route is difficult. In our case, the EAS was successfully dilated by retrograde balloon dilatation, and subsequent balloon dilatations were performed orally. Macher et al. assumed that retrograde guidewire insertion is easier than oral guidewire insertion because of the progressive narrowing of the EAS [9]. In addition, we assumed that the difficulty of oral guidewire insertion is attributed to anatomical changes. The esophageal lumen on the oral side is dilatated by saliva, and it is difficult to identify the stenotic hole. Because the esophageal lumen on the anal side of the EAS narrows progressively, the guidewire easily follows the narrowing esophagus and immediately enters the stenotic hole: the convex dome shape of the anal side of the EAS possibly allows this easy insertion.

When balloon dilatation cannot be performed, surgical procedures (such as resection of the stenotic site with or without interpositioning) are needed for EAS treatment. However, considering that these surgeries are invasive and associated with a high EAS recurrence rate, they should be considered the last treatment option (especially interposition grafting, which plays an extremely limited role in EAS treatment) [1].

In cases of EAS recurrence after dilatation, patients should be evaluated for GERD. Whether an anti-reflux surgery contributes to EAS prevention is controversial; however, some studies have shown that gastric acid contributes to EAS formation [10], whereas fundoplication for GERD prevents EAS formation [10]. However, the fundoplication method (either total or partial) that prevents EAS formation most efficiently remains unknown. In our case, partial fundoplication was performed to prevent gas bloat and dysphagia. Thus, our case indicates that partial fundoplication for GERD can cure and prevent refractory EAS.

Anti-reflux medications are another treatment option; however, the patient had already received an anti-reflux medication (i.e., proton pump inhibitors). Histamine H2 antagonists are generally considered first, but high-dose proton pump inhibitors are recommended for severe GERD [11]. Conversely, a previous report suggested that prophylactic antacid medications for patients with postoperative EA do not always prevent an EAS [12, 13]; therefore, anti-reflux surgery should be considered for treating refractory EAS with severe GERD [10]. However, the efficacy of fundoplication for EAS treatment remains unproven. One possibility is that anti-reflux medications cannot control reflux and cannot neutralize gastric acid completely. Another possibility is that non-acid reflux can contribute to EAS exacerbation. Non-acid reflux is associated with extra-esophageal symptoms such as cough at 25–50% [14] and is more correlated with a cough episode than acid reflux [15]. Non-acid reflux repeatedly stimulates the anastomotic site, resulting in an EAS. The successful treatment of an EAS with balloon dilatation may be attributed to the fact that fundoplication controls physical reflux.

The patient in our case was successfully treated with anti-reflux medication and fundoplication; however, patients in some cases may remain refractory even when GERD is corrected by fundoplication. Yasuda et al. performed a univariate regression analysis on 177 cases of EA and reported that fundoplication is a significant predictor of the need for EAS resection [16]; however, this predictor did not retain its significance in a subsequent multivariate analysis. If an EAS remains refractory after fundoplication, other treatment plans must be considered [17], including mitomycin C treatment [4, 18], endoscopic incisional therapy [19–21], and EAS resection with esophageal anastomosis [22]. Researchers have also recommended considering eosinophilic esophagitis in cases of a refractory EAS. Eosinophilic esophagitis does not respond well to dilatation unless the underlying immune mechanism is treated [23]. Kassabian et al. suggested that eosinophilic esophagitis is a frequently concomitant condition in patients with a history of esophageal deformities and that such patients may benefit from an endoscopic evaluation with biopsies [23]. Eosinophilic esophagitis should be kept in mind particularly when a patient has a history of atopy and presents with peripheral eosinophilia [24]. Our patient had no history of atopy and did not present with peripheral eosinophilia; however, we will consider this disease as a differential diagnosis in later cases. Although recurrent EASs seriously decrease the quality of life, the decline in our patient’s quality of life did not appear severe, because she could not intake food orally due to other unexplained developmental delays.

## Conclusions

From this case, we deduced that if the esophageal anastomosis site is completely obstructed on contrast examination and when oral balloon dilatation has failed, retrograde balloon dilatation for the EAS should be performed via gastrostomy. Moreover, when an EAS recurs soon after dilatation, the patient should be evaluated for GERD. If severe GERD is observed, appropriate anti-reflux surgery must be performed before dilating the EAS.

## Data Availability

Not applicable.

## Abbreviations

EAS: Esophageal anastomotic stricture
EA: Esophageal atresia
GERD: Gastroesophageal reflux disease
